# Quantification of fetal organ volume and fat deposition following *in utero* exposure to maternal Western Diet using MRI

**DOI:** 10.1371/journal.pone.0192900

**Published:** 2018-02-15

**Authors:** Kevin J. Sinclair, Lanette J. Friesen–Waldner, Colin M. McCurdy, Curtis N. Wiens, Trevor P. Wade, Barbra de Vrijer, Timothy R. H. Regnault, Charles A. McKenzie

**Affiliations:** 1 Department of Medical Biophysics, Western University, London, Ontario, Canada; 2 Department of Radiology, University of Wisconsin, Madison, Wisconsin, United States of America; 3 Robarts Research Institute, Western University, London, Ontario, Canada; 4 Department of Obstetrics and Gynaecology, Western University, London, Ontario, Canada; 5 Department of Physiology and Pharmacology, Western University, London, Ontario, Canada; 6 Children’s Health Research Institute and Lawson Health Research Institute, London, Ontario, Canada; University of Maryland School of Medicine, UNITED STATES

## Abstract

**Purpose:**

To examine the feasibility of using MRI to identify differences in liver size and fat deposition in fetal guinea pigs exposed to an *in utero* environment influenced by maternal consumption of a Western diet.

**Materials and methods:**

Female guinea pigs fed either an energy-dense Western Diet (WD), comprised of increased saturated fats and simple sugars, or a Control Diet (CD) from weaning through pregnancy, underwent MR scanning near term (~ 60 days; term ~ 69 days). Maternal weights were collected at mating and at MR scanning. T_1_-weighted, T_2_-weighted, and IDEAL water-fat images were acquired at 3 Tesla. The images were used to segment maternal adipose tissue, fetal liver, fetal brain, fetal adipose tissue, and total fetal volumes and to measure maternal and fetal hepatic fat fractions.

**Results:**

Weights of WD sows were lower prior to pregnancy (*P* = .04), however their weight gain over pregnancy did not differ from the CD group (*P* = .98). The WD sows had less total adipose tissue (TAT) at MR scanning (*P* = .04), while hepatic fat content was significantly elevated (*P* = .04). When controlling for litter size, WD fetuses had larger livers (*P* = .02), smaller brains (*P* = .01), and increased total adipose tissue volume (*P* = .01) when normalized by fetal volume. The WD fetuses also had increased hepatic fat fractions compared to CD fetal livers (*P* < .001).

**Conclusion:**

Maternal Western Diet consumption prior to and during pregnancy induces differences in maternal liver fat content, fetal liver volume and liver fat storage, as well as changes in fetal adipose tissue deposition that can be measured *in utero* using MRI.

## Introduction

Metabolic syndrome (MetS) encompasses a cluster of risk factors that, when present in combination, significantly increase the risk of diabetes and cardiovascular disease [1]. Currently in North America, approximately 20% of the population is affected by MetS, a number that is rising along with the increasingly sedentary and aging population [1, 2]. However, the rising frequency of MetS in North America, and the rest of the world, is not solely dependent on the aging population and lifestyle changes. Recent studies highlight a correlation between the risk of developing MetS and the *in utero* environment to which the offspring was exposed, where an adverse *in utero* developmental period is associated with increased metabolic disease risk later in life [3–7].

One *in utero* environment that has been associated with the development of MetS is an environment in which the fetus is exposed to a maternal Western Diet [8]. A Western Diet, sometimes referred to as a ‘fast food’ or ‘junk food’ diet, is an energy-dense diet in which fats, especially saturated fats, and simple sugars are the predominant energy sources [9]. Studies in animal models have shown that fetal exposure to a maternal Western Diet significantly increases the risk of postnatal obesity [10], hepatic steatosis and non-alcoholic fatty liver disease [11], and cardiovascular dysfunction [5]. It is also important to note that in Western society, consumption of Western-style diets is prevalent in women of reproductive age [12], with one study showing that more than half of the women in their cohort consumed more than the recommended calories from fat [12]. This observation, coupled with the above-mentioned negative impacts of poor maternal diet on the later-life metabolic health of the offspring, highlight the severity of the issue and the need for new tools in which to study the underlying physiology and clinical presentation.

The balance between fetal nutrient supply and demand has been shown to be a major factor in determining resource allocation, and in turn the propensity of human fetuses to deposit fat [13]. In an environment in which the fetus is exposed to increased levels of fatty acids and simple sugars, umbilical venous blood is preferentially shunted to the fetal liver [13]. As the fetal liver is important in fatty acid synthesis, increased nutrient availability in the liver leads to increased fetal fatty acid production which can be deposited in adipose tissue or accumulate in organs [13]. This increased fat deposition, especially in the liver, can adversely impact normal metabolic processes [11]. Over the course of gestation, this can lead to metabolic alterations in the fetus such as a reduction in the ability to oxidize fatty acids [14] as well as an increase in the propensity of the liver to store lipid [11]. These alterations may then persist into postnatal life, impacting how the offspring handles nutrients and in turn contributing to the observed increased rates of metabolic disorders associated with an increased risk of MetS in later life [13].

The current literature examining the impact of fetal exposure to maternal Western Diet has focused on the examination of offspring health after birth, but there is an unmet need for studies examining these developmental changes *in utero*. By observing abnormalities in fetal fat deposition, including increased adipose tissue deposition or increased fetal hepatic lipid content, fetuses with altered metabolism can be identified prior to birth. Detection of altered metabolism *in utero* would allow for studies to separate fetal metabolic insults from insults occurring shortly after birth, and also allow for clearer studies of *in utero* intervention potential. Furthermore, detection of altered fat deposition could help to identify fetuses that will become macrosomic, or that are at increased risk for later life disease.

Magnetic resonance imaging (MRI) is emerging as a promising imaging modality to observe fetal anatomy and physiology due to its excellent soft tissue contrast as well as its ability to measure a wide range of properties such as diffusion, perfusion, and metabolite levels via spectroscopy [15]. MRI also excels in its ability to separate fat from water, allowing for quantitative measurements of both adipose tissue volumes and fat content within organs [16]. This is particularly of interest as fetal fat deposition is thought to be altered as a consequence of fetal exposure to an environment influenced by maternal consumption of a Western Diet, and may be one of the factors leading to increased rates of MetS in later postnatal life [13].

The aim of the current study was to determine the feasibility of MRI to detect anatomical changes that signify altered metabolism in the situation of fetal exposure to a maternal Western Diet. Toward this end, guinea pig fetuses exposed to a maternal Western Diet were compared to those carried by sows consuming a Control Diet starting at weaning and through pregnancy. First, we evaluated and characterized the impact of a lifelong Western Diet exposure on the sows. Weight prior to pregnancy, weight gain over pregnancy, and whole body and hepatic fat content at MRI scan were evaluated. Second, fetal organ volumes and fat deposition were studied in order to identify anatomic differences between the two fetal groups.

## Materials and methods

### Animals and diets

All animal care and experimental procedures were carried out under a protocol approved by Western University’s Animal Care Committee in accordance with the Canadian Council on Animal Care (CCAC) standards and guidelines. Dunkin-Hartley guinea pigs (Charles River Laboratories, St-Constant, Que, Canada) were born in-house and individually housed in a temperature (20°C) and humidity (30%) controlled environment with a 12 hour light-dark cycle. The guinea pigs had free access to allocated feed and water in their cages.

**Fig 1:**
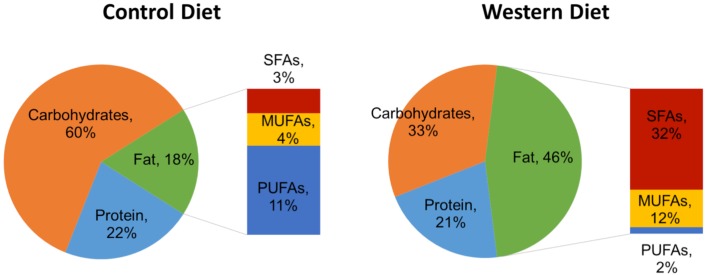
Comparison of guinea pig dietary models by macronutrient composition. Control Diet (feed caloric density = 3.4 kcal/g; left), and Western Diet (feed caloric density = 4.2 kcal/g; right). Macronutrient percentages are by calories. SFA = Saturated fatty acids; MUFA = Monounsaturated fatty acids; PUFA = Polyunsaturated fatty acids.

After birth, female guinea pigs were weaned onto either an energy-dense ‘Western Diet’ (TD: 110239, Harlan Laboratories, Madison WI) or a Control Diet (TD.110240, Harlan Laboratories), and were maintained on this diet throughout their lives. The macronutrient details of the diets are displayed in Fig 1 and their compositional details are provided in previous publications [17, 18]. Sows (F0) were mated to control males at approximately six months of age and maintained on a Control Diet or the Western Diet throughout gestation. Feed intake was recorded daily throughout the life of the sow.

### MRI

**Fig 2:**
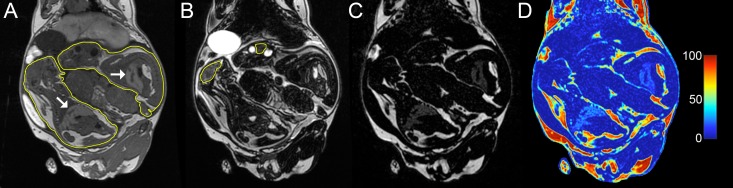
Coronal images of a pregnant guinea pig on a lifetime Western Diet. (A) T_1_-weighted image with fetuses contoured in yellow and fetal livers denoted by white arrows. (B) T_2_-weighted images with fetal brains contoured in yellow. (C) IDEAL fat-only image. (D) IDEAL fat fraction map. Images have been cropped to highlight the fetuses.

At ~60 days gestation (term ~69 days), *in vivo* MRI was performed in order to quantify maternal adipose tissue and hepatic fat content, as well as fetal organ volumes, adipose tissue volumes, and hepatic fat content. Anaesthesia was induced via induction chamber (4% isoflurane with 2 L/min O_2_) and maintained via nose cone (1.5–2.5% isoflurane with 1 L/min O_2_). Imaging was performed at 3 Tesla (MR750, GE, Waukesha, WI, USA) using a 32 element cardiac receive array (Invivo, Gainesville, FL, USA). T_1_-weighted gradient echo (repetition time/echo time [TR/TE] = 5.1ms/2.4ms, flip angle = 15°, number of averages = 4, total scan time ~ 7min; Fig 2A) and T_2_-weighted spin echo (TR/TE = 2000ms/120ms, number of averages = 2, total scan time ~ 7min; Fig 2B) images with a field of view covering the entire guinea pig were acquired with 0.875 × 0.875 mm^2^ in-plane spatial resolution and 0.9 mm slice thickness. IDEAL water-fat images (TR/ΔTE = 9.4ms/0.974ms, 6 echoes, flip angle = 4°, number of averages = 4, total scan time ~ 13min; Fig 2C and 2D) were also collected for each guinea pig with 0.933 × 0.933 mm^2^ in-plane spatial resolution and 0.9 mm slice thickness [16]. IDEAL images were accelerated with parallel MRI by a factor of 1.75 in both the phase and slice directions. Following acquisition of data, sows were recovered and returned to their cages. All of the sows recovered normally from anaesthesia and their food intake returned to normal within 24 hours. Sows pupped and resulting offspring were used in ongoing postnatal studies.

### Data analysis

Initially, 4 Control Diet (CD) and 7 Western Diet (WD) sows were scanned carrying 11 and 16 fetuses (litter size range: 2–4 and 2–3), respectively. 3 WD sows carrying 7 fetuses were excluded from the analysis due to a loss of the IDEAL water-fat data during online image reconstruction. This loss of data occurred because of processor error during image reconstruction, necessitating a system reboot before completion of data reconstruction and a complete loss of data. Final numbers for analysis were 4 CD sows and 4 WD sows, carrying 11 and 9 fetuses, respectively.

#### Sows

Maternal weights were measured at mating as well as on the MRI scan date. Using the IDEAL proton density fat fraction (PDFF) images, maternal total adipose tissue (TAT) volumes were manually segmented in Osirix (Pixmeo, Geneva, Switzerland) via connected threshold grower. IDEAL PDFF maps were also used to obtain maternal hepatic fat fraction values in Osirix by placing regions of interest (ROIs) in non-vascular regions of the liver. Multiple ROIs were used for each maternal liver and all values were averaged.

#### Fetuses

Manual volume segmentation was performed on all fetuses in ImageJ (U. S. National Institutes of Health, Bethesda, MD, USA) using a connected threshold growing algorithm and volume calculations were performed in Matlab (The Mathworks Inc., Natick, MA, USA). All manual segmentation was performed by KJS who had two years of experience. T_1_- and T_2_-weighted images were used to segment fetal liver, brain, and total fetal volumes. Brain to liver volume ratios (BLVR) were calculated by dividing the fetal brain volume by the fetal liver volume for each fetus. IDEAL PDFF images were used to segment TAT and intra-abdominal adipose tissue (IAAT) volumes for each fetus in Osirix. Hepatic fat fractions were determined by placing ROIs in non-vascular regions of each fetal liver using the PDFF maps. ROIs were obtained for multiple slices in each liver and averaged to obtain fat fraction values.

### Statistical analysis

Statistical analysis was performed using SPSS (IBM Corp., Armonk, NY, USA). For the maternal data, comparisons of means were analyzed by univariate analysis of variance (ANOVA). For the fetal data, all measured variables were tested for normality using the Shapiro-Wilk Test, and the Levene’s Test of Equality of Error Variances was performed. Since our data was normally distributed and equality of variances verified, parametric tests were utilized. A comparison of the two groups was performed using a multivariate analysis of variance (MANOVA) with litter size as a covariate to adjust for its influence on the measured variables. Litter was used as a nested factor as fetuses within a litter would have experienced a more similar environment than between litters. The dependent variables used in the analysis were: fetal volume, liver to fetal volume ratio, brain to fetal volume ratio, brain to liver volume ratio, hepatic fat fraction, TAT to fetal volume ratio, IAAT to TAT volume ratio, and IAAT to fetal volume ratio.

All comparisons between the two groups on one dependent variable were made using univariate analysis of variance with litter size as a covariate. Correlation analysis was performed using Pearson’s product-moment correlation coefficient. All data in plots comparing one group with its control are presented as box and whisker plots, with the interquartile range denoted by the box and whiskers ranging from the 5^th^ to 95^th^ percentiles. Fetal organ and adipose tissue volumes are presented as percentages of total fetal volume. For all comparisons, *P* < .05 was considered statistically significant.

## Results

### Sows and lifelong diet influence

**Fig 3:**
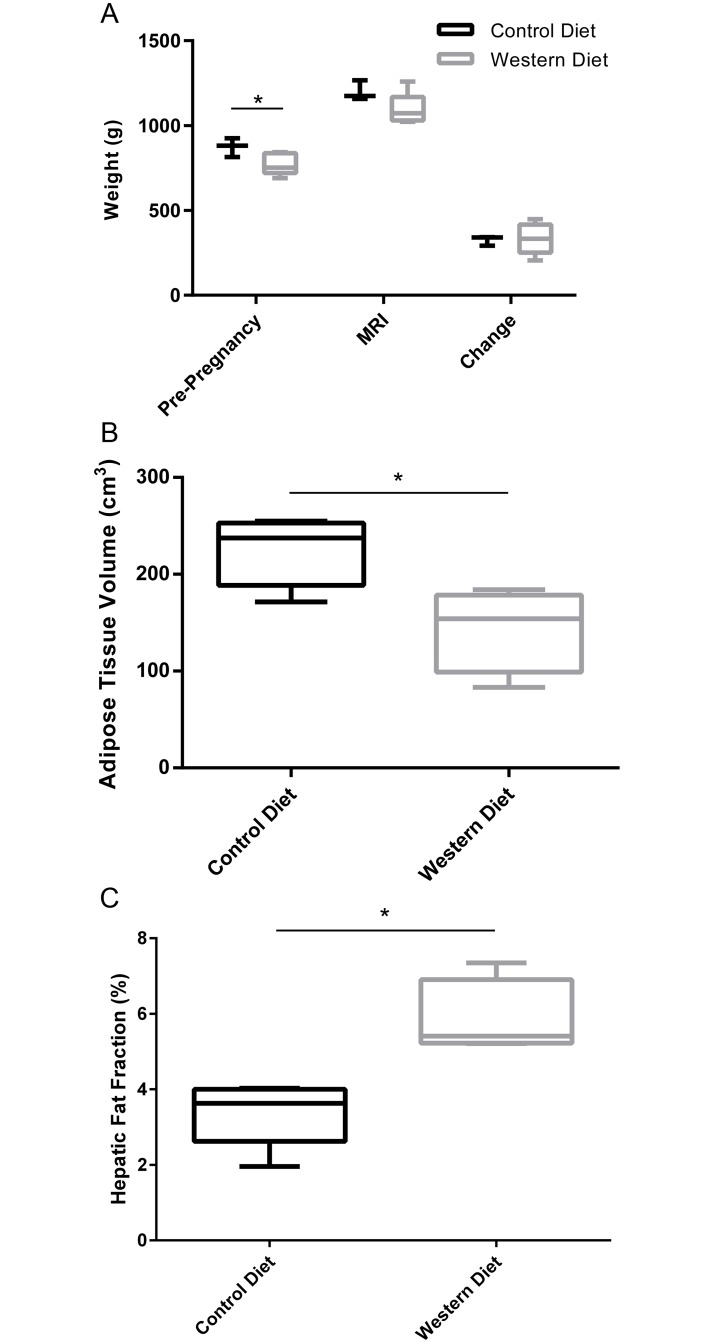
Weight and fat deposition measurements for Control Diet and Western Diet sows. (A) Weight measurements taken before pregnancy, on the MR scan date, and weight change during pregnancy (CD: *n* = 4; WD: *n* = 4). (B) Total adipose tissue volumes (CD: *n* = 4; WD: *n* = 4). (C) Hepatic proton density fat fraction (CD: *n* = 4; WD: *n* = 4). Data are presented as box and whisker plots as described in the methods section for CD (black) and WD (grey) sows. *P < .05.

From weaning to breeding, the WD sows consumed significantly more calories than the CD group (WD = 139 ± 11 kcal/day, CD = 106 ± 13 kcal/day; *P* = .005). Between breeding and MRI, the caloric intake did not differ between the two groups (WD = 149 ± 21 kcal/day, CD = 119 ± 7 kcal/day; *P* > .05). At breeding, the WD weighed significantly less than the CD (*P* = .04, Fig 3A). The WD sows still weighed less than the CD sows at MR scanning, though this result was not significant (*P* = .11), and there was no difference in weight change between the groups over the course of pregnancy (*P* = .98).

At the time of MRI, neither CD nor WD sows were overweight or obese, rather the WD sows had significantly less TAT than the CD sows (*P* = .04, Fig 3B). However, WD sows had significantly higher hepatic fat fractions than the CD sows (*P* = .04, Fig 3C).

### Fetal comparisons

Upon MANOVA, it was determined that the maternal diet had a significant effect on the measured variables (*P* < .001), and litter size was a significant covariate (*P* < .001).

#### Fetal volume measurements

**Fig 4:**
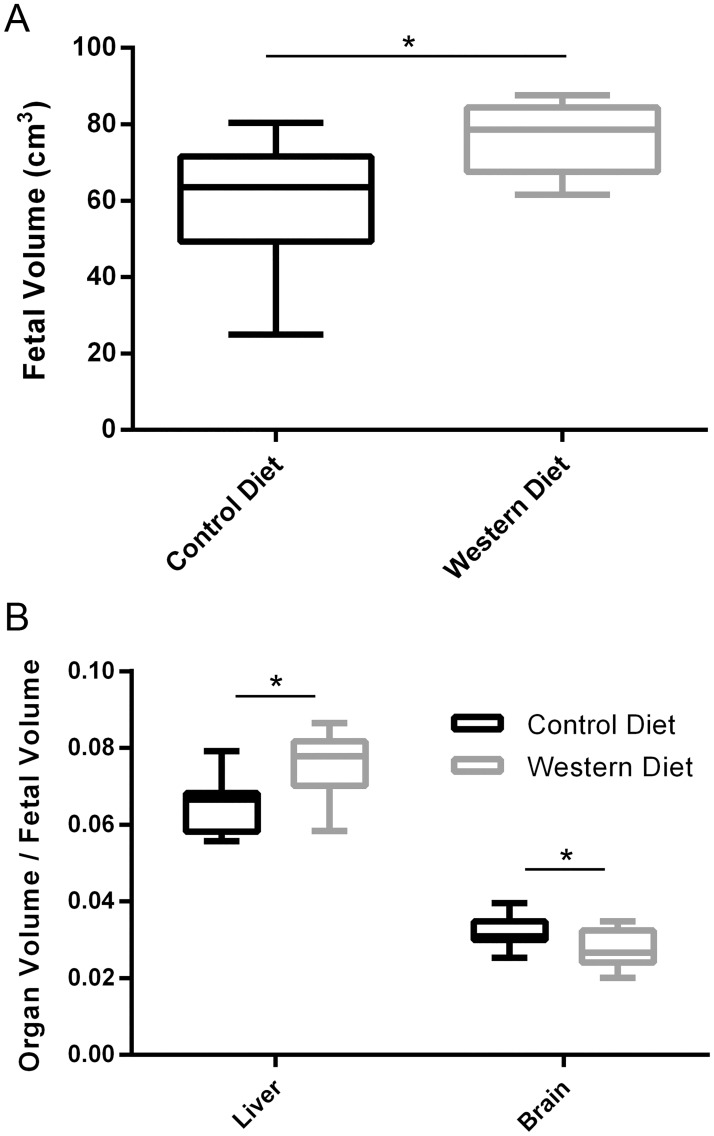
Fetal volume segmentation for Control Diet and Western Diet fetuses. (A) Total fetal volumes. (B) Liver to fetal volume ratio and brain to fetal volume ratio. Data are presented as box and whisker plots as described in the methods section for CD (black) and WD (grey) fetuses. *n* = 11 and 9 for CD and WD, respectively. *P < .05.

WD fetuses had significantly larger total fetal volumes than the CD fetuses (*P* = .007, *η*^*2*^ = .728, *power* = .933, Fig 4A). WD fetuses had significantly increased liver to fetal volume ratios than CD fetuses (*P* = .03, *η*^*2*^ = .650, *power* = .801) but decreased brain to fetal volume ratios (*P* = .02, *η*^*2*^ = .673, *power* = .846, Fig 4B), leading to a decrease in brain to liver volume ratio (0.39 ± 0.10 vs 0.49 ± 0.06, respectively, *P* < .05, *η*^*2*^ = .596, *power* = .691).

#### Fetal fat deposition

**Fig 5:**
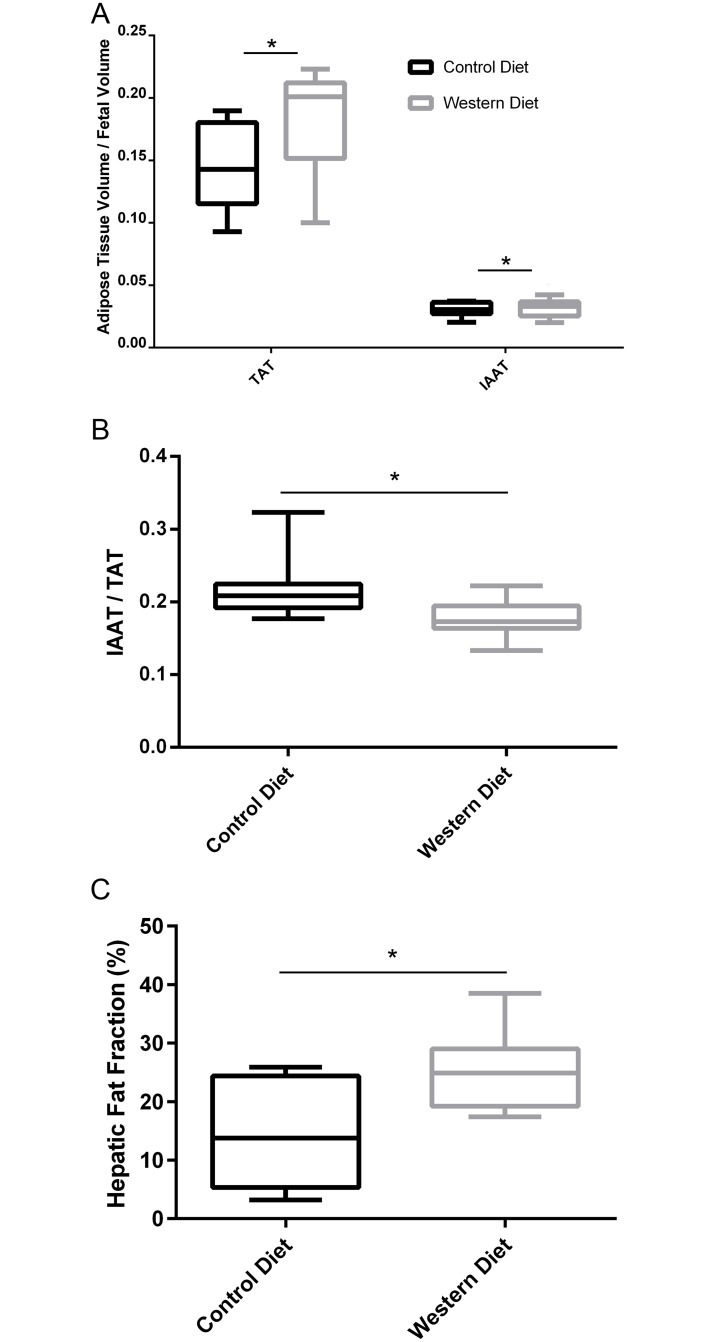
Fetal fat deposition for Control Diet and Western Diet fetuses. (A) Total adipose tissue (TAT) to fetal volume ratio and intra-abdominal adipose tissue (IAAT) volume to fetal volume ratio. (B) IAAT to TAT volume ratio. (C) Fetal hepatic proton density fat fraction. Data are presented as box and whisker plots as described in the methods section for CD (black) and WD (grey) fetuses. *n* = 11 and 9 for CD and WD, respectively. *P < .05.

WD fetuses had significantly greater TAT to fetal volume ratios than the CD group (*P* < .001, *η*^*2*^ = .968, *power* = 1.00), as well as an increase in IAAT to fetal volume ratios (*P* = .01, *η*^*2*^ = .705, *power* = .900, Fig 5A). Even though both TAT to fetal volume ratio and IAAT to fetal volume ratio were increased, WD fetuses had significantly less IAAT to TAT ratios (*P* < .001, *η*^*2*^ = .862, *power* = 1.00, Fig 5B). Finally, the WD fetuses displayed increased hepatic fat content compared to controls (*P* < .001, *η*^*2*^ = .861, *power* = 1.00, Fig 5C).

**Fig 6:**
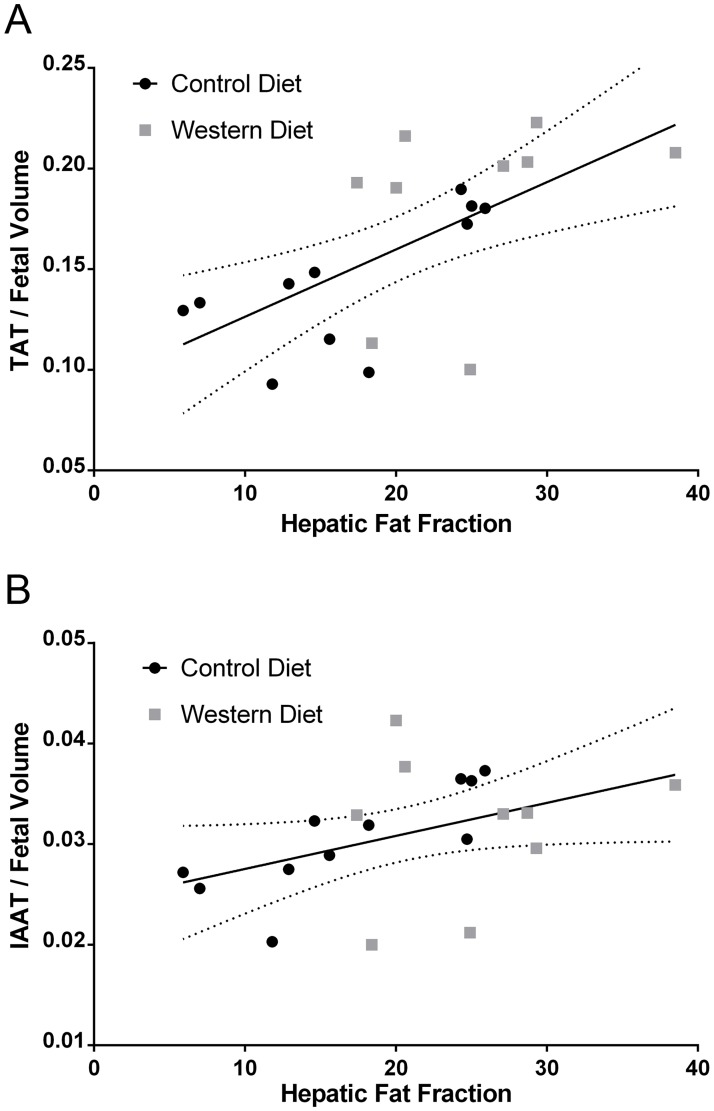
Correlation of fetal adipose tissue volumes with hepatic fat fraction. (A) Total adipose tissue (TAT) to fetal volume ratio as a function of fetal hepatic proton density fat fraction (PDFF). (B) Intra-abdominal adipose tissue (IAAT) to fetal volume ratio as a function of fetal hepatic PDFF. Linear fit lines for all data are shown with 95% confidence intervals.

We further examined the correlation of liver PDFF with fetal TAT to fetal volume ratio (Fig 6A) as well as IAAT to fetal volume ratio (Fig 6B). It was found that TAT to fetal volume ratio correlated well with hepatic PDFF (*R*^2^ = .39, *P* = .003) while IAAT to fetal volume ratio did not correlate strongly (*R*^2^ = .19, *P* = .06). Interestingly, the correlation of TAT to fetal volume ratio and IAAT to fetal volume ratio with hepatic PDFF was much stronger in the CD group (*R*^*2*^ = .47, *P* = .02 and *R*^*2*^ = .63, *P* = .004, respectively) than the WD group (*R*^*2*^ = .12, *P* = .36 and *R*^*2*^ = .01, *P* = .05, respectively).

## Discussion

Fetal exposure to a maternal Western Diet is associated with long-term metabolic health risk for the offspring [5–7]. However, little is known about the *in utero* manifestations that bring about this increased metabolic disease prevalence in postnatal life. In the current study, we sought to evaluate the utility of MRI in the examination of potential anatomical changes *in utero* that highlight metabolic alterations arising due to exposure to a WD. We evaluated this utility by examining the ability to identify differences in fetal brain and liver volumes, as well as fetal fat deposition in guinea pigs born to sows on either a life-long energy-dense WD or a CD.

Sows that had consumed the WD since weaning and through pregnancy were not significantly heavier than CD sows and they had less adipose tissue, despite consuming more calories on a daily basis since weaning. One potential cause of this reduction in adipose tissue, despite the elevated fat content of the WD, is the increase of fructose in the WD compared to the CD. Studies in rats have reported decreased weight and adipose tissue in offspring fed a high-fructose diet compared to those consuming a CD [19–21], and high-fructose diets have been shown to reduce the ability of adipose tissue depots to synthesize fatty acids [22]. Further, high-fructose diets have been reported to promote the release of free fatty acids from adipose tissue, which then accumulate in the serum and ultimately in organs such as the liver [23, 24]. This proposed mechanism of increased liver fat accumulation could also explain the increase in hepatic fat fraction in the WD sows observed in the current report, although this is an exciting area of research requiring further investigation.

Many studies examining the effects of fetal exposure to a Western-like Diet in rodent models did not observe a significant effect on birth weight [5, 14, 25, 26], but failed to take litter size into account. We used litter size as a covariate, resulting in the detection of a significant increase in fetal volume in the WD fetuses versus CD fetuses. When controlling for litter size, fetal guinea pigs of sows on a WD also have altered growth compared to those maintained on a CD. This is observed most notably in the fetal guinea pig’s propensity to store fat, especially in the liver. This result complements the idea proposed by Godfrey *et al*. [13] that exposure of the placenta to excess fatty acids and simple sugars should lead to an increase in nutrients to the fetal liver resulting in increased *de novo* lipogenesis. Since guinea pig fetuses, similar to human fetuses, do not start to develop significant adipose tissue stores until late gestation [27, 28], excess lipid delivered to and produced by the fetal liver earlier in gestation would preferentially be stored in the liver and other organs [11]. Multiple studies on non-alcoholic fatty liver disease (NAFLD) in humans have shown that increased hepatic lipid levels are linked with increased rates of metabolic and cardiovascular disease [6, 29, 30]. It could be postulated that increased hepatic lipid storage at such an early stage of development could put the individual at an even greater risk to develop liver metabolic abnormalities following birth due to an increase in time of exposure.

There was an observed decrease in brain to fetal volume ratio in the WD group compared to the CD group. Though seemingly counterintuitive, this was mainly a result of the noted increase in fetal volume, as the absolute fetal brain volumes did not differ between groups. The WD fetuses had larger liver to fetal volume ratios than the CD fetuses. This result was also observed in a study comparing 3 week old male rat offspring born to mothers consuming a high-fructose, high-saturated fat diet [19]. It has been proposed that increased nutrient transfer to the fetal liver takes place in response to increased placental exposure to fatty acids and simple sugars [13]. Elevated nutrient supply to the fetal liver has previously been shown to increase cell proliferation in the fetal organs. This could provide an explanation for the increased fetal liver to fetal volume ratios, though further study is required [31]. Further, the WD fetuses displayed increased fetal liver fat fraction compared to the CD fetuses. This is consistent with a previous study on the effects of a high fat diet on nonhuman primate fetuses [11].

The increased TAT to fetal volume ratios in the WD fetuses in the current study are consistent with the increased body fat content observed at birth in guinea pigs born to sows on a high-fat diet [26] as well as rats born to mothers on a “junk-food” diet [32]. Similarly, increased fetal fat content was also observed *in utero* in diabetic human pregnancies [33]. We also observed an increase in IAAT to fetal volume ratio in the WD group, though this difference was smaller than the difference seen in the TAT to fetal volume ratio, leading to a decrease in the ratio of IAAT to TAT. This signifies a preference of the WD fetuses to store excess adipose tissue subcutaneously. An interesting outcome of the correlation analysis was that normalized adipose tissue volumes were more strongly correlated with hepatic fat fraction in the CD group compared to the WD group. More study is necessary to understand this outcome, but it could speak to the complex nature of allocating excess nutrients in the developing fetus.

In the current study, the feasibility of MRI in detecting differences in fetal growth was examined. To accomplish this, an examination of the effects of a maternal WD on fetal organ growth and fat deposition in guinea pigs was performed. A quantitative IDEAL sequence was used for water-fat separation as it provides reliable water-fat separation and an accurate quantitative measurement of proton density fat fraction [16]. The validation of adipose tissue volume assessment with MRI has previously been reported in humans [34] and small animals [35], and the IDEAL technique has been previously validated in a small animal model by comparing measured proton density fat fraction measurements to lipid extraction fraction [36].

The use of the guinea pig presents a number of strengths for the type of studies outlined above. Fetal guinea pig development is very similar to that of humans, as their development mainly occurs prenatally [26, 37]. Further, unlike other rodents, guinea pig fetuses develop significant fat stores, with both humans and guinea pigs being born with approximately 15% fat by body weight, while rats and mice are born with < 2% [26]. This model for fetal growth, while a good model, presents some limitations. First, guinea pigs seldom present as singleton pregnancies. This increases the variability of *in utero* environment severity within a single group as the sharing of resources is determined by multiple factors. Multiple fetuses within a single pregnancy may have different nutrient availabilities and thus outcomes compared with a singleton pregnancy [38]. In this study, we chose to use litter size as a covariate in order to eliminate some of this variability from our analysis. Additionally, guinea pigs develop *in utero* with extremely elevated hepatic fat content compared to humans [39, 40], with guinea pigs reported to have up to 25% fat by wet weight [40] and normal human fetuses having essentially no hepatic fat [41]. This may affect direct translation of our hepatic fat fraction results to human fetuses, though we hypothesize that when exposed to a WD, human fetuses will similarly show a relative increase in hepatic fat levels. This hypothesis is supported by two previous studies that showed a correlation between neonatal hepatic lipid levels and maternal body mass index (BMI) [42, 43]. A final limitation of the study was that, as the fetuses were allowed to pup naturally, the sex of each pup was not matched to the imaging data. Thus, we could not include fetal sex as a covariate in our analysis. However, it has been shown that birth phenotype is not sex dependent in guinea pigs [44] and thus may not have a significant influence on our results.

In this feasibility study, we have shown that fetal MRI is capable of detecting developmental differences between fetuses exposed to a maternal WD, and those who developed *in utero* in sows on a CD. While the results in this study on their own do not directly show a metabolic programming effect on the fetuses, these findings, especially the WD fetuses’ increased lipid accumulation, are indicative of altered metabolic programming. Further, a growing body of literature exists in support of the hypothesis that maternal nutrition and obesity can affect offspring metabolic outcomes such as increased adiposity [45, 46], insulin sensitivity [19], blood pressure [47], and non-alcoholic fatty liver disease [48, 49]. Further study is now warranted to examine these differences on a larger scale in order to verify our preliminary results, as well as to examine how the macroscopic differences observed relate to metabolic alterations *in utero*, and how they may influence increased rates of obesity and MetS in later life. Additionally, we feel that these methods described can also easily be translated to human study and clinical practice, as our MRI scanning was performed on a clinical system using human radiofrequency coils. Finally, similar methods may be applied to other suboptimal *in utero* environments such as in intrauterine growth restriction, where stunted growth of the fetus is linked to later life metabolic and cardiovascular disease similar to fetuses exposed to the Western Diet [50].

In conclusion, we have shown that MRI is capable of detecting differences in growth between fetal guinea pigs born to sows on an energy-dense WD and those on a CD, independent of maternal obesity. The most important difference observed was in the fetus’s propensity to store fat in the liver and adipose tissue, as increased lipid deposition, especially in organs and intra-abdominally, is an outcome associated with metabolic and cardiovascular disease. The results of this study show the value of MRI in the study of adverse *in utero* environments that are associated with later life MetS.

## Supporting information

S1 Table Segmentation results. (DOCX)

S1 Fig Example adipose tissue segmentation images. Example images showing fetal TAT segmentation (left) and maternal TAT segmentation (right).
(TIF)
