# Supplementary material for: Quantification of fetal organ volume and fat deposition following in utero exposure to maternal Western Diet using MRI
Source: PLoS One. 2018 Feb 15;13(2):e0192900. doi: 10.1371/journal.pone.0192900 (PMC5814025; doi:10.1371/journal.pone.0192900)
Supplement: S1 Table — (DOCX) [file pone.0192900.s001.docx]

**Table 1 – Weight and segmentation results for maternal guinea pigs**

|  | Diet | Litter Size | Pre-Pregnancy Weight (g) | Weight at MRI (g) | Segmented Adipose Tissue volume (cm^3^) | Hepatic Fat Fraction |
| --- | --- | --- | --- | --- | --- | --- |
| *Sow 1* | CD | 3 | 815 | 1157 | 251 | 3.98 |
| *Sow 2* | CD | 2 | 881 | 1174 | 255 | 3.29 |
| *Sow 3* | CD | 2 | 926 | 1267 | 171 | 1.96 |
| *Sow 4* | CD | 4 |  |  | 206 | 5.30 |
| *Sow 5* | WD | 3 | 690 | 1030 | 83 | 5.25 |
| *Sow 6* | WD | 2 | 734 | 1023 | 146 | 5.21 |
| *Sow 7* | WD | 2 | 843 | 1260 | 184 | 5.57 |
| *Sow 8* | WD | 2 | 752 | 1085 | 162 | 7.35 |

**Table 2 – Segmentation results for fetal guinea pigs**

|  |  | Diet | Fetal Volume  (cm^3^) | Fetal Liver Volume (cm^3^) | Fetal Brain Volume (cm^3^) | Fetal Hepatic Fat Fraction (%) | Fetal TAT Volume (cm^3^) | Fetal IAAT Volume (cm^3^) |
| --- | --- | --- | --- | --- | --- | --- | --- | --- |
| *Sow 1* | *Fetus 1* | CD | 78.8 | 4.59 | 1.99 | 11.8 | 7.32 | 1.6 |
|  | *Fetus 2* | CD | 59.6 | 3.45 | 1.83 | 18.2 | 5.89 | 1.9 |
|  | *Fetus 3* | CD | 80.4 | 4.48 | 2.58 | 15.6 | 9.27 | 2.32 |
| *Sow 2* | *Fetus 1* | CD | 28.2 | 2.76 | 1.5 | 3.2 | 1.32 | 0.24 |
|  | *Fetus 2* | CD | 63.8 | 5.05 | 2.26 | 14.6 | 9.47 | 2.06 |
| *Sow 3* | *Fetus 1* | CD | 56.3 | 3.84 | 2.23 | 12.9 | 8.04 | 1.55 |
|  | *Fetus 2* | CD | 63.3 | 4.5 | 1.79 | 5.9 | 8.2 | 1.72 |
| *Sow 4* | *Fetus 1* | CD | 57.8 | 3.92 | 2.01 | 7 | 7.71 | 1.48 |
|  | *Fetus 2* | CD | 68.9 | 4.58 | 2.13 | 25 | 12.5 | 2.5 |
|  | *Fetus 3* | CD | 68.0 | 3.99 | 2.06 | 24.3 | 12.9 | 2.48 |
|  | *Fetus 4* | CD | 69.9 | 4.16 | 2.1 | 25.9 | 12.6 | 2.61 |
| *Sow 5* | *Fetus 1* | WD | 83.8 | 6.3 | 1.89 | 29.3 | 18.68 | 2.48 |
|  | *Fetus 2* | WD | 78.6 | 6.8 | 1.58 | 38.5 | 16.34 | 2.82 |
|  | *Fetus 3* | WD | 87.6 | 7.14 | 2.24 | 28.7 | 17.8 | 2.9 |
| *Sow 6* | *Fetus 1* | WD | 67.9 | 3.96 | 2.36 | 24.9 | 6.8 | 1.44 |
|  | *Fetus 2* | WD | 74.0 | 6.07 | 1.97 | 18.4 | 8.38 | 1.48 |
| *Sow 7* | *Fetus 1* | WD | 85.1 | 6.62 | 2.53 | 20.6 | 18.4 | 3.21 |
|  | *Fetus 2* | WD | 80.3 | 6.44 | 2.7 | 17.4 | 15.5 | 2.64 |
| *Sow 8* | *Fetus 1* | WD | 61.6 | 4.63 | 1.62 | 27.1 | 12.4 | 2.03 |
|  | *Fetus 2* | WD | 67.2 | 4.37 | 2.1 | 20 | 12.8 | 2.84 |
