# Supplementary figures and images for: Quantification of fetal organ volume and fat deposition following in utero exposure to maternal Western Diet using MRI
Source: PLoS One. 2018 Feb 15;13(2):e0192900. doi: 10.1371/journal.pone.0192900 (PMC5814025; doi:10.1371/journal.pone.0192900)

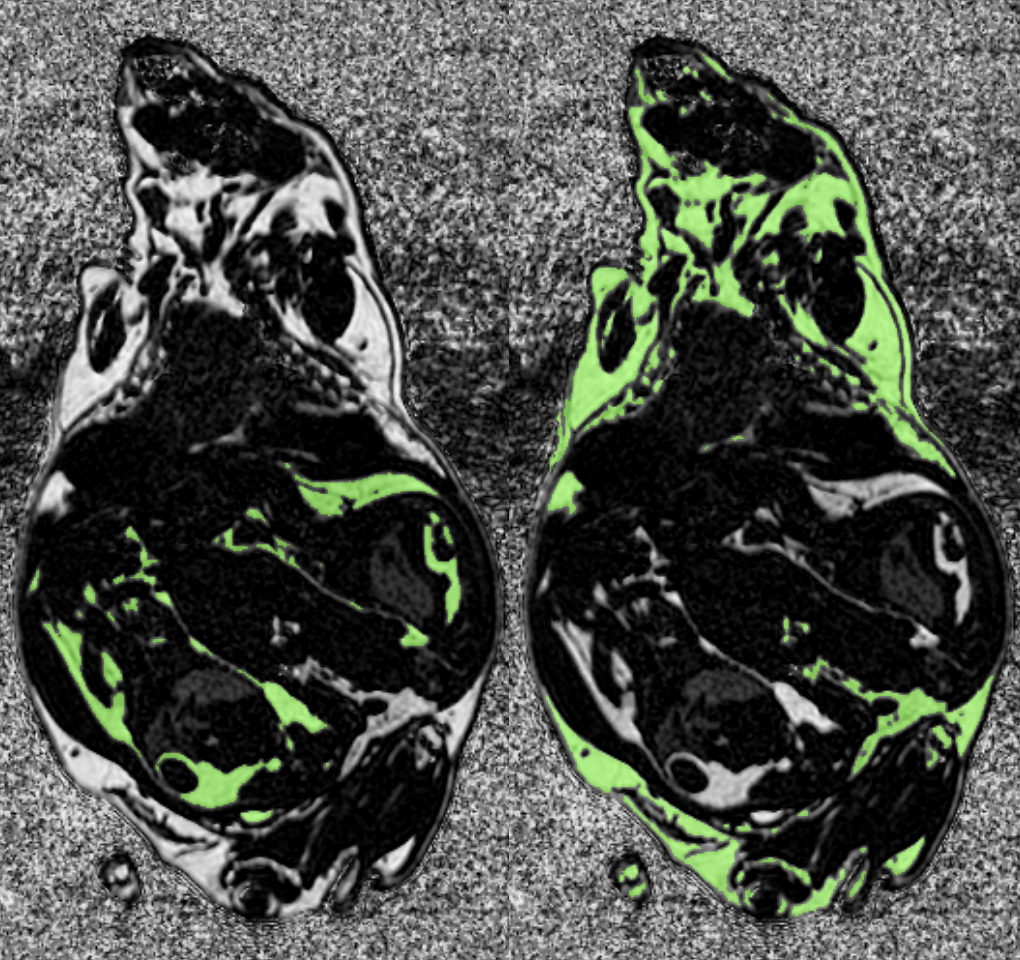

Supplement: S1 Fig — Example images showing fetal TAT segmentation (left) and maternal TAT segmentation (right). (TIF) [file pone.0192900.s002.tif]
